# Spinal anaesthesia with Chloroprocaine HCl 1% for elective lower limb procedures of short duration: a prospective, randomised, observer-blind study in adult patients

**DOI:** 10.1186/s12871-021-01279-9

**Published:** 2021-02-20

**Authors:** Daniela Ghisi, Giorgia Boschetto, Alessandra Maria Spinelli, Sandra Giannone, Jacopo Frugiuele, Marcello Ciccarello, Stefano Bonarelli

**Affiliations:** 1 Anesthesia, Intensive Care and Pain Therapy, Istituto Ortopedico Rizzoli, via G.C. Pupilli 1, 40136 Bologna, Italy; 2 Institute of Anesthesiology and Intensive Care, Azienda Ospedaliera di Padova-Universita’ degli Studi di Padova, via C. Battisti 267, 35128 Padova, Italy; 3 Anesthesia, Intensive Care and Pain Therapy, Ospedale G. e C. Mazzoni, via degli Iris 1, 63100 Ascoli Piceno, Italy; 4 Anesthesia, Intensive Care and Pain Therapy, Dipartimento Rizzoli-Sicilia, Istituto Ortopedico Rizzoli, SS 113 al Km 246, 90011 Bagheria, Italy

**Keywords:** Ambulatory, subspecialties, Anaesthetics, local, Chloroprocaine, Neuraxial blocks: spinal, regional anaesthesia, Transient neurologic symptoms, complications

## Abstract

**Background:**

This prospective, randomised, observer-blinded study has been conducted in patients undergoing procedures of the lower extremities to evaluate the time to complete block resolution of 2-chloroprocaine 1% at three intrathecal doses (30, 40 and 50 mg).

**Methods:**

After informed consent, we enrolled 45 male and female patients, aged 18–65 years, ASA score I-II, BMI 18–32 kg/m^2^, undergoing elective lower limb procedures lasting ≤40 min and with a requested dermatomeric level of sensory block ≥ T12.

The patients were randomised in a 1:1:1 ratio to receive Chloroprocaine HCl 1% at one of the three different intrathecal doses (Group 30 = 30 mg, Group 40 = 40 mg or Group 50 = 50 mg). The progression and regression of both sensory and motor blocks were evaluated blindly. Urine and venous blood samples were collected for pharmacokinetic analysis.

**Results:**

Times to regression of spinal blocks were 1.76 ± 0.35 h, 2.13 ± 0.46 h and 2.23 ± 0.38 h, in Group 30, 40 and 50 respectively: the 30 mg dose showed a significantly faster resolution of spinal block than the 40 mg (*p* = 0.034) and the 50 mg (*p* = 0.006). Time to readiness for surgery was significantly reduced with the dose of 50 mg when compared to dose of 30 mg (*p* = 0.0259).

**Conclusions:**

The doses of 50 mg and 40 mg yielded a longer resolution of spinal block than the dose of 30 mg. Nevertheless, the dose of 30 mg resulted in a higher secondary failure rate.

**Trial registration:**

Registration of clinical trial: clinicaltrials.gov (NCT02481505).

## Introduction

Clinical studies in volunteers investigated chloroprocaine use for spinal anaesthesia at doses ranging between 30 and 60 mg [1, 2]. The dose of 2-chloroprocaine in clinical studies mainly varies from 40 to 50 mg [3–5]. Previous dose-finding studies showed that 30 mg was associated with an insufficient duration of analgesia for surgical procedures over 60 min or more and requiring a dermotomeric level of anaesthesia ≥ T10, whereas time to complete block resolution was significantly prolonged when a dose of 50 mg is used [6]. Goldblum and Atchabahian recommended the dose of 30 mg for surgery of the lower extremities up to 40–60 min duration [7].

We therefore conducted this prospective, randomized, observer-blind study in adult patients to reevaluate the efficacy of chloroprocaine HCl 1% conservative free solution at the doses previously investigated (30, 40 and 50 mg) for spinal anaesthesia in shorter procedures of the lower limbs.

The present study also served as a phase II clinical study for the Food and Drug Administration (FDA) approval of the intrathecal use of 2-chloroprocaine 1%. The study inlcudes the analysis of the pharmacokinetic profile of the drug after intrathecal administration to confirm the low systemic exposure to chloroprocaine following spinal injection.

## Materials and methods

This was a prospective, single centre, randomised, parallel-group, observer-blind, three doses, efficacy and pharmacokinetic study. The study was registered at clinicaltrials.gov (NCT02481505, 25/06/2015). The study **have** received approval by an independent Ethics Committee (Ethics Committee, Istituto Ortopedico Rizzoli di Bologna - IRCCS, Bologna on 23rd of February 2015). All methods were carried out in accordance with relevant guidelines and regulations (Declaration of Helsinki). After written informed consent, we enrolled 45 male and female patients, aged 18–65 years, classified with an American Society of Anaesthesiologists’ (ASA) physical status score I-II, with a body mass index (BMI) of 18–32 Kg/m^2^, undergoing elective lower limb procedures lasting ≤40 min and with a requested dermatomeric level of sensory block ≥ T12.

We excluded patients with contraindications to spinal anaesthesia, ASA physical status III-IV, ascertained of presumptive hypersensivity to the active principle or formulations ingredients, a history of neuromuscular diseases or with significant history of medical conditions that could interfere with the aim of the study (including diabetes and other neuropathies), a history of drug or alcohol abuse, pregnant or lactating women and patients with chronic pain syndromes (taking opioids, antidepressants, anticonvulsivant agents or chronic analgesic therapy).

The patients were randomised to one of the three treatment groups in a 1:1:1 ratio (Group 30, Group 40, Group 50) to receive Chloroprocaine HCl 1% at one of the three different intrathecal doses (Group 30 = 30 mg, Group 40 = 40 mg or Group 50 = 50 mg corresponding to 3, 4 and 5 ml, respectively) according to the randomised, parallel-group design of the study. The randomisation list was computer-generated by the Biometry Unit at the Contract Research Organization (CRO) using the PLAN procedure of the SAS® system version 9.3. Once in the anesthesia induction room, an intravenous (i.v.) line was placed in all the patients and midazolam 0.03 mg/kg was administered i.v. for sedation. Vital signs (non-invasive blood pressure (NIBP), three-lead electrocardiography (ECG) and pulse oximetry (SpO_2_) were recorded at the screening visit, at baseline in the operating room before administering the spinal block and then every 10 min from spinal injection until block resolution. At baseline, the following normal ranges for haemodynamic variables were used: sysotlic blood pressure (SBP): 100–139 mmHg, diastolic blood pressure (DBP): 50–89 mmHg, heart rate (HR): 45–90 beats/min. Occurrence of clinically significant hypotension (defined as a decrease in SBP by approximately 30% or more from baseline values) and bradycardia (defined as a HR decrease below 45 bpm) were monitored throughout the study and, if observed, appropriately treated.

The spinal injection was performed with a Whitacre 25 Gauge needle in lateral decubitus at the intervertebral space L3/L4 or L4/L5 with a midline apporach and the needle bevel oriented towards the upper surgical side. The progression of both sensory and motor blocks was evaluated by an observer blinded to the dose and volume administered, with a Pinprick test and Bromage’s scale, every 2 min until readiness for surgery, then every five minutes until the achievement of the maximum level of sensory block and the regression of at least two dermatomes. After that, the assessment of sensory and motor blocks was performed every 30 min until complete resolution of spinal block (recovery of motor function and sensory block at S1). Readiness for surgery was defined as the association of an adequate motor block (Bromage score ≥ 2, Tmb) and the loss of Pinprick sensation at the dermatomeric level of T12 (Tsb).

Venous blood samples for pharmacokinetic (PK) analysis were collected from a forearm vein within 60 min before investigational medicinal product (IMP) administration (T0) and 5 (T5), 10 (T10), 30 (T30) and 60 (T60) minutes after IMP administration. Six ml of blood were collected into heparinised tubes (Na-heparin) containing a mix of esterase inhibitors. The tubes were gently inverted for 4–5 times and then put in a bath of water and ice. Then, within 15 min from collection, the samples were centrifugated at 4 °C for 10 min at 200 rounds per minute (RPM) to obtain plasma. Each plasma sample was immediately divided into two aliquots of about 0.5–1 ml each (P1 and P2) in pre-labelled polypropylene tubes and immediately put in a bath of water and ice. Then, within 15 min, the tubes were stored frozen at − 70 °C until analyses.

The start and end times of the surgical procedures were recorded. During surgery, each spinal block was considered adequate if neither adjunctive analgesia nor sedation were necessary, inadequate if rescue anaesthesia or analgesia (up to fentanyl 2 μg/kg iv in bolus) were required or failed if general anaesthesia was necessary to complete the procedure.

The regression of sensory block was defined with the Pinprick test (using a 20-G hypodermic needle) as the restoration of sensory perception at the level of S1. The regression of motor block was defined as the return to a Bromage’s score = 0. After surgery, non-steroidal anti-inflammatory drugs and paracetamol were administered to treat postoperative pain, if needed. If pain continued, the nurses were allowed to administer either tramadol or oral morphine or the association paracetamol/tramadol according to the anaesthesiologist’s indication. We also recorded the time to unassisted deambulation, to first spontaneous urine voiding and to eligibility for home discharge (fulfillment of a score ≥ 18 in the modified Aldrete’s score). The first postoperative urine was collected into containers, its volume was measured and, after through mixing, two aliquots of 0.1 mL each (U1 and U2) were prepared in polypropylene tubes. The two aliquots were stored at − 70 °C until analyses. If the collected urine could not be immediately processed, the complete sample was kept refrigerated at approximately 4 °C before measurement of volume and preparation of the two aliquots.

Adverse events were investigated, with particular attention to signs and symptoms of local toxicity, any sign of hypersensitivity reaction or haemodynamic instability and to the occurrence of transient neurologic symtoms (TNS), defined as paraesthesia of the lower limbs and buttocks or back pain radiating to the legs, without sensory or motor deficit, resolved spontaneously within few days. Eventual adverse events and TNS were also investigated at 24 h and 7 ± 1 days after surgery with two follow-up phone calls. Patients were questioned for fatigue, nausea/vomiting, dizziness, urination/defecation difficulty, pain at the site of injection and at the site of surgery, unusual sensations such as burning, tingling, dull, aching, numbness, hypoaesthesia.

For pharmacokinetic (PK) analysis, the concentration of 2-Chloroprocaine was determined in plasma and the concentration of the metabolite 2-chloro-4-aminobenzoic acid (CABA) was determined in plasma and urine at Accelera laboratories (Accelera S.r.l., Viale Pasteur, 10 - 20014 Nerviano (MI) - Italy) using a validated liquid chromatography tandem mass spectrometry (LC-MS/MS) method that can detect both analytes to a lower quantification limit (LQL) of 4 ng/mL for plasma and 50.0 ng/ml for urine. Analyses were performed according to the general Principles of “OECD Good Laboratory Practices for testing of chemicals” and to the FDA guideline for the validation of bioanalytical methods.

### Statistical analysis

Forty-five male/female patients (15 patients per dose group) were planned. The primary outcome of the study was time to end of anaesthesia (Tea). Secondary outcomes were the onset and other offset times as well as pharmacokinetic variables. Results from a previous study of spinal injection of Chloroprocaine HCl 1% [6] have been taken in consideration to calculate the required study sample size and normal distribution of data had been assumed. Sample size was calculated using n-Query Advisor 7.0. When the sample size in each of the 3 doses groups is 13, a one-way analysis of variance would have 80% power to detect a difference in time to complete spinal block regression (Tea) means at the 0.05 level, characterized by a variance of means (where G = 3) of 249.962, assuming that the common standard deviation is 30.01. The sample size was increased of about 15% in order to be more conservative. Fifteen patients were therefore enrolled and treated per each dose group.

Continuous variables were summarised by dose level group using classic descriptive statistics (i.e. mean, SD, CV%, min, median and max) while categorical variables were summarised by dose level group using tables of frequencies. Due to the small sample size, collected data were compared using nonparametric tests (Kruskal-Wallis test, Wilcoxon rank-sum test for paiwise comparison).

Regarding the PK analysis, data below the lower quantification limit (BLQL) were considered as 0 and presented as BLQL in listings and tables. As a consequence of BLQL (i.e. 0) values, calculated geometric means (if requested) could be null. For this reason, in the presence of any null value, the geometric mean was reported as not calculated (NC). Urinary excretion of CABA was calculated as the amount of metabolite excreted as a percentage of the administered dose (molar ratio).

## Results

**Fig. 1:**
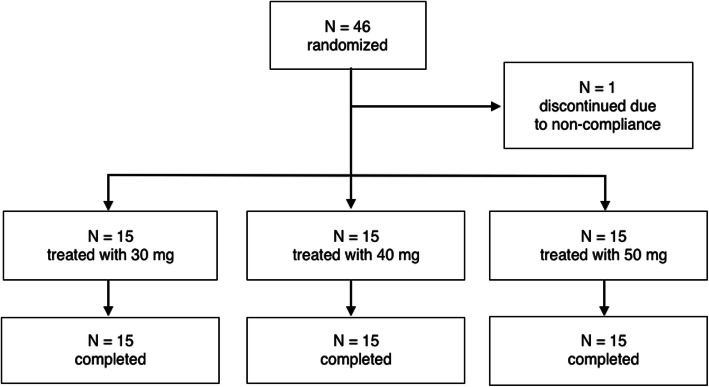
The investigator included 46 subjects in the study. One subject (S030/029) discontinued the study before receiving the assigned anaesthesia due to non-compliance. Among the remaining 45 patients, 15 enrolled subjects received chloroprocaine 30 mg, other 15 received chloroprocaine 40 mg, and other 15 received chloroprocaine 50 mg. Forty-five subjects completed the study as planned

The investigator included and randomized in the study 46 subjects between June 2015 and November 2015. Disposition of subjects is summarized in Fig. 1. After randomization, 45 subjects received the study treatment, as planned. As per study desing, 15 enrolled subjects received 30 mg, other 15 received 40 mg, and other 15 received 50 mg of 2-Chloroprocaine 1%.

One subject (S030/029) was discontinued from the study before receiving the assigned anaesthesia due to non-compliance. Forty-five (45) subjects completed the study as planned (Fig. 1).

Demographic data (median, range, SD and frequency data) of the study analysis sets are presented in Table 1.

**Table 1:** Anthropometric characteristics and duration of surgery in the three groups of patients (Group 30 mg, Group 40 mg, Group 50 mg of Chloroprocaine 1%). Data are expressed as median (range), mean ± SD and frequency (%) according to data distribution

	FAS (45 patients)		
**Dose Group**	**Chloroprocaine 1%**		
	Group 30 mg	Group 40 mg	Group 50 mg
**Gender**			
Female n (%)	6	6	6
Male n (%)	9	9	9
**Age (years)**			
Mean ± SD	40.8 ± 12.1	39.4 ± 12.5	41.5 ± 13.9
Median (range)	42 (22–58)	41 (19–58)	40 (10–63)
**Body weight (kg)**			
Mean ± SD	73.27 ± 14.11	70.27 ± 13.32	74.07 ± 13.14
Median (range)	78 (47–99)	66 (52–99)	73 (48–98)
**Height (cm)**			
Mean ± SD	172 ± 6.9	170.2 ± 9.3	168.9 ± 7.1
Median (range)	170 (158–185)	173 (153–185)	170 (153–180)
**BMI (kg/m**^**2**^**)**			
Mean ± SD	24.65 ± 3.96	24.19 ± 3.62	25.87 ± 3.71
Median (range)	25.3 (18.8–30.6)	22.2 (19.0–30.6)	25.8 (18.3–31.3)
**Race**			
Mestizo n (%)	1 (6.7%)	1 (6.7%)	0
White n (%)	14 (93.3%)	14 (93.3%)	15 (100%)

The times of complete regression of the spinal block (Tea) for the doses 30, 40 and 50 mg of Chloroprocaine were 1.76 ± 0.35 h, 2.13 ± 0.46 h and 2.23 ± 0.38 h, respectively. The 30 mg dose showed a significantly faster resolution of spinal block than the 40 mg (*p* = 0.034) and the 50 mg (*p* = 0.006) in the full set analysis (FAS).

The times to onset of sensory and motor blocks, times to readiness for surgery and to the maximum level of sensory block are represented in Table 2. Time to readiness for surgery was significantly reduced with the dose of 50 mg when compared to dose of 30 mg (*p* = 0.0259).

**Table 2:** Time to onset/offset variables. Data are expressed as mean ± SD. Tsb: time to onset of sensory block, Tmb: time to onset of motor block, Trs: time to readiness for surgery, Tsbmax: time to maximum level of sensory block, Trd: time to regression of two dermatomes; TS1: time to regression of sensory block to S1, Tea time to regression of both motor and sensory blocks; Trmb: time to regression of motor block; Tua: time to unassisted ambulation; Tra: time to first administration of rescue analgesia; Tuv: time to first spontaneous urinary voiding; Thd: time to home discharge

	FAS			
Dose Group	**Chloroprocaine 1%**			
	Group 30 mg	Group 40 mg	Group 50 mg	*p* value
Tsb (min)	5.4 ± 3.0	6.6 ± 3.4	4.8 ± 2.0	NS
Tmb (min)	6.3 ± 3.2	6.0 ± 3.3	4.4 ± 2.3	NS
Trs (min)	8.0 ± 4.1	7.9 ± 4.7	5.3 ± 2.0	30 vs 50 mg p = 0.0259
Tsbmax (h)	0.22 ± 0.14	0.24 ± 0.1	0.23 ± 0.10	NS
Trd (h)	0.687 ± 0.361	0.851 ± 0.468	0.695 ± 0.290	NS
TS1 (h), Tea (h)	1.761 ± 0.348	2.127 ± 0.457	2.195 ± 0.386	30 vs 50 mg p = 0.0094 30 vs 40 mg p = 0.0344
Trmb (h)	1.438 ± 0.409	1.480 ± 0.400	1.661 ± 0.459	NS
Tua (h)	2.662 ± 0.789	3.361 ± 1.120	3.213 ± 0.856	30 vs 40 mg *p* = 0.0471
Tra (h)	0.717 ± 0.397	0.315 ± 0.049	–	NS
Tuv	2.530 ± 0.761	3.361 ± 1.120	3.067 ± 0.755	30 vs 50 mg p = 0.0412 30 vs 40 mg p = 0.0171
Thd	3.021 ± 1.012	3.545 ± 1.281	3.530 ± 0.887	NS

The proportion of patients reaching an effective anaesthesia with an adequate spinal block increased with the dose. Neither rescue anaesthesia nor rescue analgesia were required when the 50 mg dose was administered while 3 patients (20%) in Group 30 and 2 patients (13.3%) in Group 40 required additional fentanyl or sedation to complete surgery. No patient required general anaesthesia to complete the surgical procedure. It took 0.72 ± 0.40 h for the three patients in Group 30 and 0.32 ± 0.05 for the two patients in Group 40 to ask for additional analgesia or anaesthesia intraoperatively.

Time to regression of two dermatomes of the maximum level, time to resolution of motor block, time to unassisted ambulation, time to resolution of sensory block, time to first analgesic request, time to urinary voiding, time to readiness for home discharge are represented in Table 2. Time to resolution of sensory block was faster in Group 30 than in Group 50 both in the PP set (p = 0.0259) and in the FAS (*p* = 0.0094), as well as in Group 30 than in Group 40 in the FAS (*p* = 0.0344). Time to urinary voiding was faster in Group 30 compared to Group 40 (*p* = 0.0412) and to Group 50 (*p* = 0.0171) in the FAS. No patients required urinary catheterisation.

Maximum levels of sensory block are reported in Table 3. No statistically significant difference between treatments was detected.

**Table 3:** Maximum metameric level of sensory block and overall comparison, n (%) is shown

	FAS			
Dose Group	**Chloroprocaine 1%**			
	Group 30 mg	Group 40 mg	Group 50 mg	p value
T2 n (%)	0	2 (13.3)	2 (13.3)	*p* = 0.2951
T3 n (%)	1 (6.7)	0	1 (6.7)	
T4 n (%)	3 (20.0)	2 (13.3)	1 (6.7)	
T6 n (%)	0	1 (6.7)	4 (26.7)	
T7 n (%)	1 (6.7)	0	1 (6.7)	
T8 n (%)	3 (20.0)	3 (20.0)	2 (13.3)	
T10 n (%)	3 (20.0)	1 (6.7)	3 (20.0)	
T12 n (%)	3 (20.0)	5 (33.3)	1 (6.7)	
L1 n (%)	1 (6.7)	1 (6.7)	0	

Mean ± SD plasma concentrations of chloroprocaine and CABA are shown in Table 4.

**Table 4:** Pharmacokinetic variables: plasma concentrations (ng/ml) of chloroprocaine and CABA. Mean ± SD is shown; BLQL: below the quantification limit (4.0 ng/ml)

	FAS					
Dose Group	**Chloroprocaine 1%**			**CABA**		
	30 mg	40 mg	50 mg	30 mg	40 mg	50 mg
Pre-dose (0)	BLQL	BLQL	BLQL	BLQL	BLQL	BLQL
5′ post-dose	BLQL	BLQL	BLQL	16.13 ± 20.11	20.41 ± 23.04	24.89 ± 20.34
10′ post-dose	BLQL	BLQL	BLQL	41.44 ± 31.78	38.85 ± 25.49	75. 83 ± 67.64
30′ post-dose	BLQL	BLQL	BLQL	57.46 ± 43.77	67.18 ± 36.90	97.65 ± 61.70
60′ post-dose	BLQL	BLQL	BLQL	47.02 ± 41.38	53.09 ± 31.80	78.38 ± 48.40

**Fig. 2:**
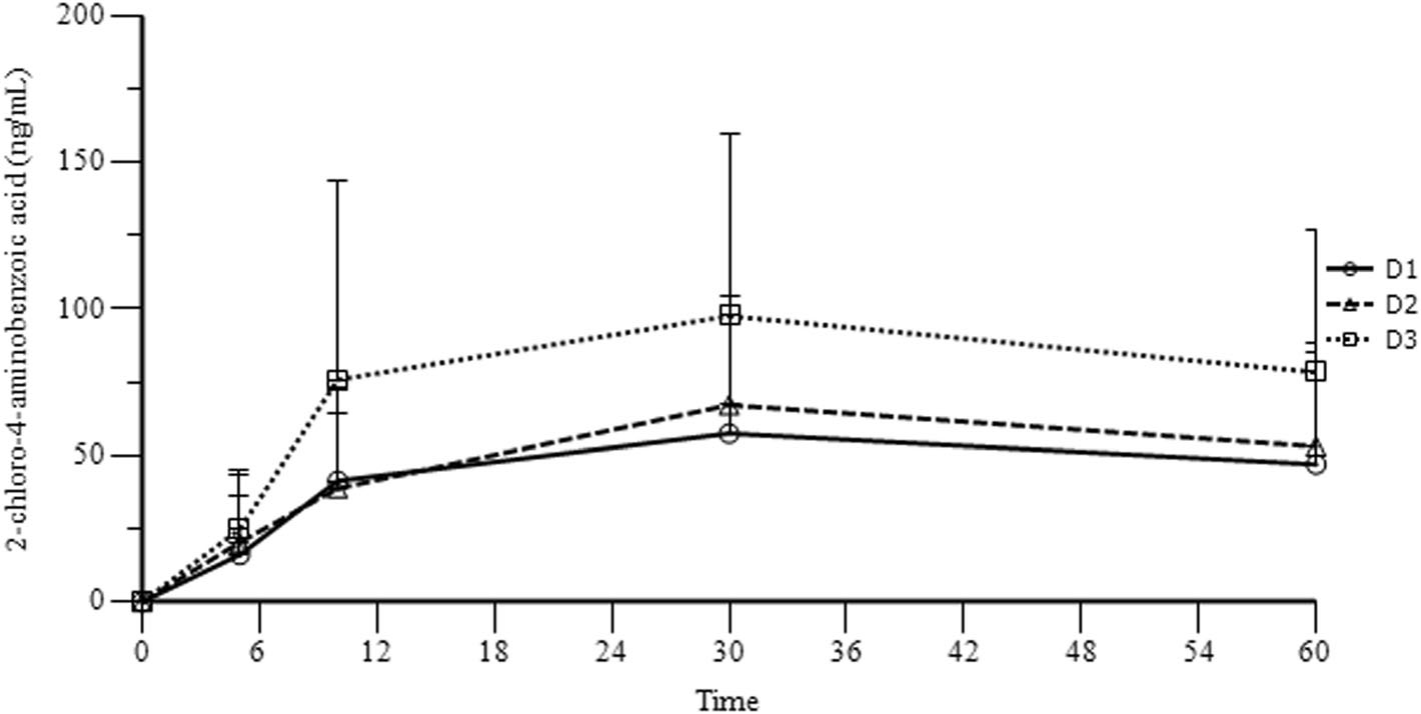
CABA increased in plasma after the spinal injection of the parent compound and reached a peak 30 min post-dose. Plasma CABA concentrations increased with the increase in chloroprocaine dose

Chloroprocaine was not quantifiable in any plasma sample of any patient. On the contrary CABA was quantifiable in most plasma samples. CABA increased in plasma after the spinal injection of the parent compound and reached a peak 30 min post-dose (Fig. 2). Plasma CABA concentrations increased with the increase in the chloroprocaine dose.

Mean ± SD urine concentrations of CABA were comparable among groups (Table 4): 1217.7 ± 627.8 ng/ml in Group 30 vs 1603.3 ± 1827.9 ng/ml in Group 40 vs 1679.6 ± 2103.5 ng/ml in Group 50. The amount of excretion of CABA was also similar in the three groups: 1.70 ± 0.97% in Group 30 vs 1.76 ± 0.93% in Group 40 vs 1.66 ± 1.29% in Group 50.

The most frequent adverse event registered in the study population was postoperative pain at the site of surgery which occurred in 93% of patients receiving the 30 mg dose, 87% of patients in he 40 mg dose and 87% of patients in the 50 mg dose. The second most frequent adverse event was postoperative pain at the site of spinal injection (18% of the study population). Paracetamol was administered for pain at the site of surgery in 100% of patients in Group 30, 80% of patients in Group 40 and 67% of patients in Group 50. Ketoprofen was administered to 53% of patients in Group 30, 47% of patients in Group 40 and 60% of patients in Group 50. No patient in Group 40 required a second-step opioid pain rescue medication, while 47 and 60% of patients in Group 30 and 50 respectively requested a second-step analgesic (either tramadol, oral morphine or the association between paracetamol and tramadol). No difference in time to first analgesic request was registered postoperatively among the three groups (*p* = 0.9567).

Among the other adverse events, no clinically significant variations values of vital signs were observed during the study with 2 exceptions. Subject S007/007 suffered from a transient mild bradycardia, 22 min after the spinal injection of chloroprocaine 40 mg. The episode lasted for one minute and was counteracted with an i.v. injection of atropine. Subject S004/004 was suffering from hypertension at study entry and therefore SBP and DBP values measured at screening, baseline and discharge were out of range, but due to the underlying disease. No patient experienced significant hypotension as defined in the protocol and at the time intervals described in the protocol.

No patient reported any TNS at either telephonic follow-ups. Neither moderate nor severe adverse events were reported.

## Discussion

The present prospective, randomized, blind-observer study evaluated the efficacy and the tolerability of preservative-free Chloroprocaine HCl 1% at the doses of 30, 40 and 50 mg.

As expected, the time to end of anaesthesia (Tea) on average coincided with the resolution of sensory block (TS1). The doses of 50 mg and 40 mg yielded a Tea and a TS1 significantly longer than those yielded by the dose of 30 mg. In the past, the spinal use of chloroprocaine in the US was largely described in doses ranging between 20 and 60 mg [8, 9]. In 2011 the use of this range of doses of chlororpocaine 1% was reported effective and safe in surgeries lasting 38 ± 23 min, registering a mean time to ambulation of 155–207 min and an incidence of primary and secondary block failure of 1.2 and 0.8%, respectively [10]. Previous investigation in the same setting with surgeries of the lower extremity, lasting between 45 and 60 min, suggested that even if a dose of 30 mg provides a faster regression of spinal block than either 40 or 50 mg, it results in a higher secondary failure rate, without accelerating home discharge [6]. Our results are consistent with previous literature. In fact, reducing the duration of surgery to 40 min and restricting indication to surgeries requiring a T12 level of anaesthesia in the present study allowed us to achieve a higher success rate with the lower doses of 30 and 40 mg than in the previous study by Casati et al. [6] Although the proportion of patients reaching an effective anaesthesia with an adequate spinal block increased with the IMP dose from 80% in group 30 mg to 100% after the 50 mg dose, some of these patients reguiring additional analgesia/anaesthesia underwent longer procedures than expected (42 min in one case and 1 h and 14 min in the other), confirming that the smaller dose of 30 mg fits surgeries for which a shorter duration is foreseen.

Time to unassisted ambulation (Tua) showed a statistically significant difference between 30 and 40 mg. No difference was found between 30 and 50 mg in terms of Tua, which both yielded on average a Tua shorter than the dose of 40 mg. This conflicting result may be explained by the fact that Tua depends on many different events beyond recovery of motor block, such as the patient’s readiness to stand up and walk with crutches after surgery, the availability of the floor nurse to help patient at the first mobilization, possible side effects after the procedure, such as lightheadedness or post-operative nausea and vomiting. Despite the significant difference in terms of Tea, time to readiness to home discharge was not different among groups: within an average time ranging between 3 and 3.5 h patients in all groups were ready for home discharge. Readiness for home discharge was investigated in our study every 30 min: a shorter time frame for registration of the same parameter could have detected a significant difference among groups, although without clinical relevance.

Chloroprocaine showed a favourable pharmacokinetic profile at all the three tested doses. As expected, chloroprocaine was not quantifiable in sieric plasma after the spinal injection, whereas the metabolite CABA was quantifiable in most plasma samples. Plasma CABA concentrations increased in plasma after the spinal injection of the parent compound and reached a peak 30 min post-dose, showing proportionality to the correspondent increase in chloroprocaine dose. The percentage amount of excretion of CABA in the first postoperative urine collection was approximately 1.70% with all three doses (30, 40 and 50 mg). Chloroprocaine is rapidly metabolized in the plasma by hydrolysis of the ester linkage by the enzyme pseudocholinesterase with the production of two major metabolites, i.e. β-diethylaminoethanol and 2-chloro-4-aminobenzoic acid (CABA), which inhibits the action of the sulfonamides [11]. Only few PK studies have been performed of local anaesthetics administered intrathecally [11–13] and some understanding comes from computational models of cerebrospinal fluid dynamics. To our knowledge, no studies on the PK of chloroprocaine or its metabolite CABA after intrathecal injection have been performed.

A study of Chloroprocaine 2 and 3% in obstetric patients after epidural anaesthesia [14] showed that in maternal plasma chloroprocaine was detectable for 5–10 min after each dose (doses: 468 ± 284 mg for vaginal delivery and 948 ± 347 mg for caesarean section, respectively, administered through an epidural catheter). Mean chloroprocaine levels were 51 ± 13 (range 0–470) and 23 ± 80 (range 0–335) ng/mL for vaginal delivery and caesarean section, respectively. Chloroprocaine levels of 2.7 ± 0.7 ng/mL were found in maternal blood at delivery in a study of chloroprocaine administered by continuous infusion [14]. In another study performed by the same authors in 1982, chloroprocaine levels of 10.0 ± 1.5 (detected in 8/50) and of 12.05 ± 1.7 ng/mL (detected in 2/30 subjects) were reported [15]. It is assumed that the plasma concentrations of chloroprocaine after spinal injection are much lower than after epidural administration, also considering that approximately 10–30 times lower doses are administered intrathecally. In geriatric patients, for example, the absorption of another local anaesthetic, i.e. lidocaine 2%, after spinal injection was much lower than after epidural administration [12].

In our small population, chloroprocaine showed an excellent safety profile at all the three tested doses. Indeed, no adverse event related to chloroprocaine or the spinal injection was reported and all patients showed hemodynamic stability. In general, no moderate nor severe adverse events occurred during the study. No TNS were registered at the two telephonic follow-ups performed at 24 h and 7 days after surgery.

In conclusion, the dose of 30 mg showed a slower onset and a faster offset than the 50 mg dose, without affecting time to readiness for home discharge. Moreover the dose of 30 mg yielded an effective anaesthesia in the 80% of patients as compared to the 50 mg dose which was effective in the 100% of patients. Restricting the duration of surgery and the dermatomeric extension fo the spinal block in the present study yeldied a better success rate than in previous literature, [6] confirming that the smaller dose of 30 mg fits surgeries for which a shorter duration is foreseen.

The sieric plasma measurement of chloroprocaine, along with the urine and plasmatic measurement of CABA evidenced the absence of the parent drug in the blood stream and the presence of CABA in both plasma and urine. Two-chloroprocaine 1% had an excellent safety profile at all the three tested doses. Indeed, no adverse event related to chloroprocaine nor TNS occurred during the study.

The FDA considered that this study contributed with adequate data to recommend approval (September 26, 2017) of the 50 mg dose of 2-chloroprocaine 1% (10 mg/mL) for the indication of single intrathecal injection in adults.

## Data Availability

The datasets used and/or analysed during the current study are available from the corresponding author on reasonable request. All data generated or analysed during this study are included in this published article.
